# Not Just an Oil Slick: How the Energetics of Protein-Membrane Interactions Impacts the Function and Organization of Transmembrane Proteins

**DOI:** 10.1016/j.bpj.2014.04.032

**Published:** 2014-06-03

**Authors:** Sayan Mondal, George Khelashvili, Harel Weinstein

**Affiliations:** † Department of Physiology and Biophysics, Weill Cornell Medical College of Cornell University, New York, New York; ‡ The HRH Prince Alwaleed Bin Talal Bin Abdulaziz Alsaud Institute for Computational Biomedicine, Weill Cornell Medical College of Cornell University, New York, New York

## Abstract

The membrane environment, its composition, dynamics, and remodeling, have been shown to participate in the function and organization of a wide variety of transmembrane (TM) proteins, making it necessary to study the molecular mechanisms of such proteins in the context of their membrane settings. We review some recent conceptual advances enabling such studies, and corresponding computational models and tools designed to facilitate the concerted experimental and computational investigation of protein-membrane interactions. To connect productively with the high resolution achieved by cognate experimental approaches, the computational methods must offer quantitative data at an atomistically detailed level. We show how such a quantitative method illuminated the mechanistic importance of a structural characteristic of multihelical TM proteins, that is, the likely presence of adjacent polar and hydrophobic residues at the protein-membrane interface. Such adjacency can preclude the complete alleviation of the well-known hydrophobic mismatch between TM proteins and the surrounding membrane, giving rise to an energy cost of residual hydrophobic mismatch. The energy cost and biophysical formulation of hydrophobic mismatch and residual hydrophobic mismatch are reviewed in the context of their mechanistic role in the function of prototypical members of multihelical TM protein families: 1), LeuT, a bacterial homolog of mammalian neurotransmitter sodium symporters; and 2), rhodopsin and the β1- and β2-adrenergic receptors from the G-protein coupled receptor family. The type of computational analysis provided by these examples is poised to translate the rapidly growing structural data for the many TM protein families that are of great importance to cell function into ever more incisive insights into mechanisms driven by protein-ligand and protein-protein interactions in the membrane environment.

## Introduction

Experimental evidence for the participation of the membrane in the function and organization of various transmembrane proteins has been accumulating for well over three decades (1–3). Such evidence continues to be collected for diverse protein families, including cell surface receptors like GPCRs (4–7) and nicotinic acetylcholine receptors (2,3), for ion channels like the mechanosensitive (8) and potassium channels (9), and for transporters like SERCA (10) the Na^+^, K^+^-ATPase (1), and the sodium-coupled secondary symporters (11–13). However, for such complex proteins, the quantitative biophysical characterization of the membrane interactions underlying the identified effects on function and spatial organization has lagged behind, both methodologically and conceptually. In this mini-review, we describe recent progress in quantifying such interactions and their mechanistic consequences, achieved from biophysical analysis and computational modeling (14–18). In particular, we focus on progress in attaining a quantitative mechanistic understanding of lipid-protein interactions at a detailed molecular level that parallels that achieved by advanced experimentation in molecular biophysics. To enable direct comparison to results from such high-resolution experiments, the mechanistic predictions from the theoretical and computational studies must include the identification of specific residues and structural motifs of the complex proteins that are responsible for the mechanistically relevant protein-membrane interactions and the resulting energy components.

A key consideration in the energetics of protein-membrane interaction, which also has the potential of yielding detailed information about the role of specific structural elements, is the well-known phenomenon of hydrophobic mismatch (HM), i.e., the mismatch between the hydrophobic thickness of a protein and that of the unperturbed membrane in which it is embedded (17,19–21). In fact, the experimental observations regarding the membrane-dependence of the function and/or organization of the various transmembrane proteins mentioned above pointed to the involvement of the HM and its determination by membrane thickness (1,3,6,9,20,21). An example is the observation from Förster resonance energy transfer (FRET) measurements that the visual receptor rhodopsin oligomerizes to different extents in membranes composed of lipids with different tail lengths (6). The information regarding specific structural elements can be extracted from the specific role of the HM in determining mechanistic aspects of membrane protein function, through the energy cost of exposing structural elements of the embedded protein to unfavorable environments (e.g., of hydrophobic residues to water).

The energy cost of the HM can be alleviated, in principle, by membrane deformations that would reduce the exposure of the affected structural elements to the unfavorable environments (20,22–24). However, from the increasingly detailed information gained on the structural and dynamic properties of the multi-TM proteins in the classes discussed above, it becomes clear that the extent of such alleviation of HM by membrane deformation is susceptible to the conformation and organizational state (e.g., monomer versus oligomer) of the proteins (14–16,18), because their different conformations and organization states have different lipid-protein interfaces. Mechanistically, this means that the HM phenomenon drives a compromise between the energy cost of membrane deformation and the energetics of various conformational and organizational states of the protein in the membrane, thereby modulating the distribution among the energetically different states of both the membrane and the proteins embedded in it.

**Figure 1:**
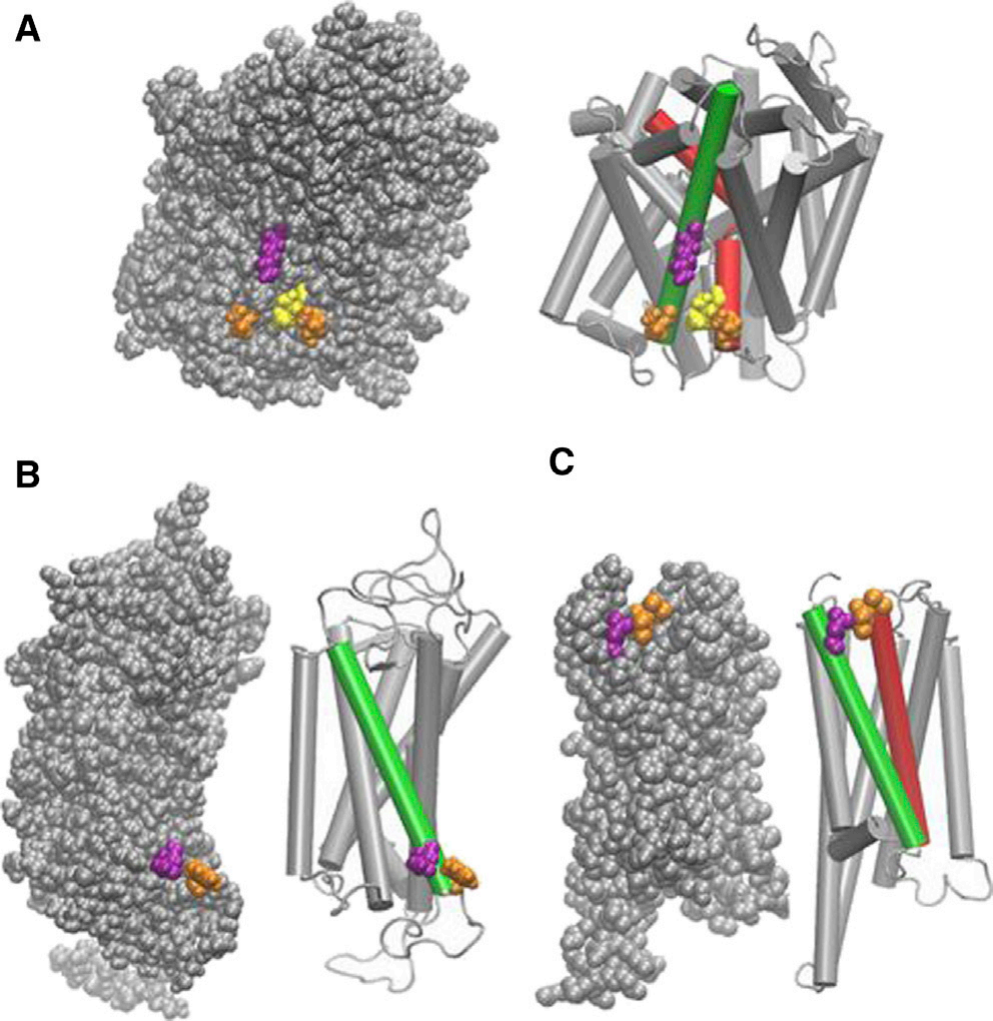
Examples of adjacent polar (*purple*) and hydrophobic (*orange*) residues on the membrane-facing surface of multi-TM proteins. The residues identified below are highlighted on the proteins shown in both van der Waals representation and cartoon representation with the key TMs highlighted. (*A*) A snapshot from an MD simulation of Leucine transporter (LeuT) in its occluded conformation (see Mondal et al. (18)), highlighting the adjacency of the charged K288 of TM7 (*green* TM) to the hydrophobic residues L12 in TM1 (*red*) and L277 of TM7 (*green*). Also highlighted is residue I15 (*yellow*), which becomes more membrane-facing with conformational change from the occluded to the inward-open conformation (see Table 1). (*B*) A snapshot from an MD simulation of rhodopsin (see Mondal et al. (14,17)), showing the juxtaposition of the polar Q5.60 and the hydrophobic F5.63, both in TM5 (*green*). (*C*) Snapshot from an MD simulation of the dopamine D2 receptor, showing the polar N1.33 in TM1 (*green*) adjacent to the hydrophobic V2.66 and V2.67 residues in TM2 (*red*) (17). (Panels *B* and *C* are adapted with permission from Mondal et al., 2013 (17). Copyright by Springer.) To see this figure in color, go online.

The HM phenomenon is conceptually simple, but the complexity of the membrane proteins of interest in the classes mentioned above makes its use in the evaluation of the energy cost of membrane deformation, and with that the identification of the mechanistically critical structural elements, somewhat more difficult. One complication is that the mode and extent of protein exposure to the membrane will change with the conformational rearrangements and protein-protein interactions associated with the function of these proteins. But even structurally, they present a challenge for the evaluation of the energy cost of the HM, because these proteins usually consist of multiple transmembrane segments (TMs), which vary in the lengths and tilts of their hydrophobic portions. The radial asymmetry of the hydrophobic surface of the entire protein becomes a critical consideration in the evaluation of the HM energy cost (see Fig. 1 and the literature (14,15,18)), because the combined interface of these proteins with the surrounding membrane often contains regions where polar and hydrophobic residues are located next to one another. Such adjacencies of residues with different polarity properties in the proteins comprising multiple TM segments (termed here “multi-TM proteins”) are given in Fig. 1 with examples from several multi-TM proteins representing common structural folds of functionally important families.

Fig. 1 *A* shows an adjacency in the bacterial leucine transporter LeuT (18), which has the prototypical fold of sodium symporters in the NSS class that includes the neurotransmitter transporters for serotonin, dopamine, and norepinephrine (11,25–28). In LeuT, the membrane-facing hydrophobic residues L12 in TM1, and L277 in TM7, are shown in Fig. 1 *A* to be adjacent to a membrane-facing, positively charged residue, K288 from TM7 (18). Fig. 1 *B* shows a similar type of adjacency in the structure of a prototypical Class A G-protein coupled receptor (GPCR), the visual receptor rhodopsin that has long served as a model system for studying lipid-protein interactions in GPCRs (4,6,7,29–34). Here the polar residue Q5.60 is adjacent to the hydrophobic residue F5.63 in TM5 (to enable direct comparison of structure-related location of corresponding residues in hundreds of other GPCRs of the same fold, GPCR residues are identified here with the Ballesteros-Weinstein generic numbering system in which the index *n.xy* marks the position of a residue in the *n*th TM relative to the most conserved residue in that TM, which is given the number 50 (35)). Fig. 1 *C* presents an example from another GPCR, the dopamine D_2_ receptor, where the polar N1.33 residue is adjacent to the hydrophobic V2.66 and V2.67 residues (17).

**Figure 2:**
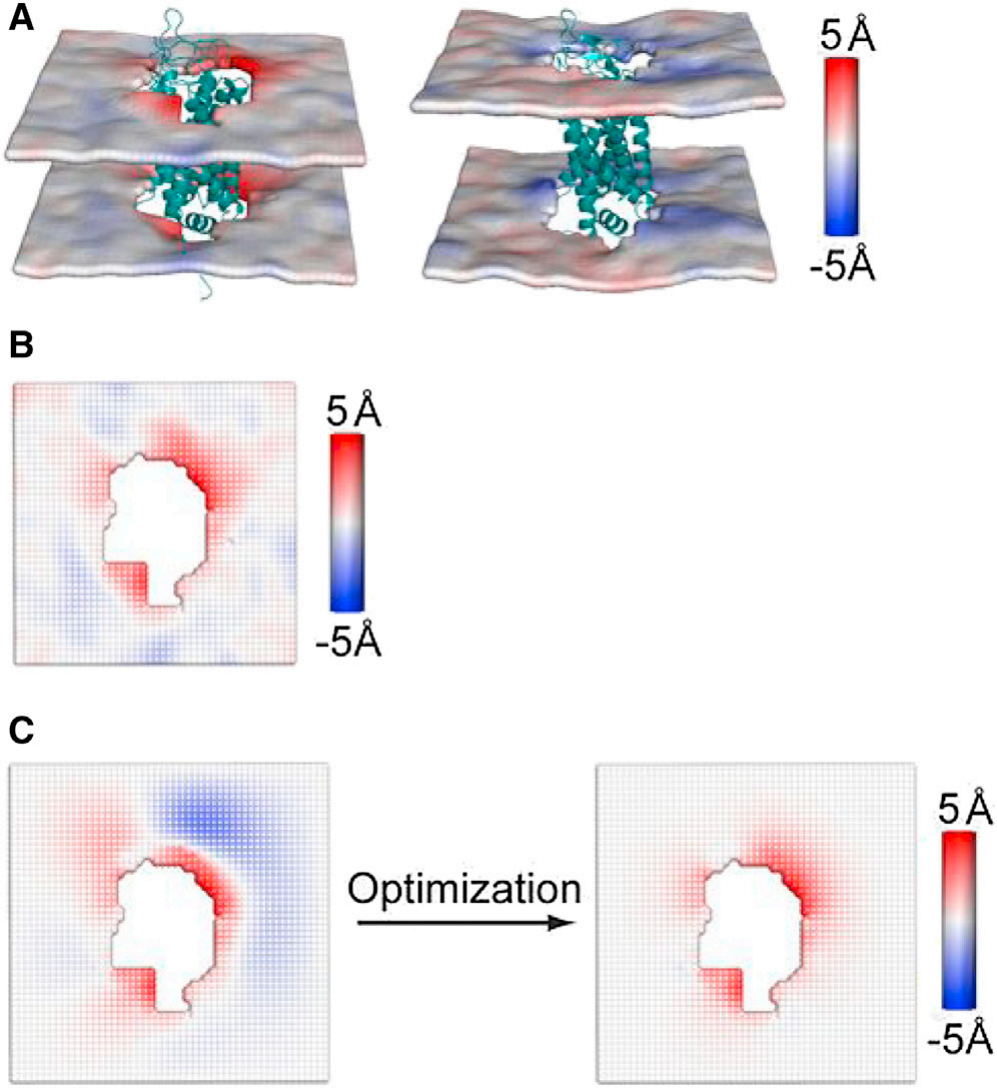
Radially asymmetric membrane deformations around rhodopsin. (*A*) Deformation profiles *u*(*x*,*y*) of the membranes of monounsaturated 14-Carbon di(C14:1)PC (*left panel*) and monounsaturated 20-Carbon di(C20:1)PC (*right panel*) around rhodopsin calculated directly from the MD simulations (14). The deformation profile, shown as a color map projected onto the membrane surface, was obtained by fitting a grid (spacing 2 Å) to the positions of the phosphate atoms in the two leaflets during the trajectory, followed by time-averaging and spatial smoothing. (*B* and *C*) Application of CTMD to compute the energetics of membrane deformations for rhodopsin in di(C14:1)PC. Panel *B* shows *u*(*x*,*y*) calculated directly from the MD simulation trajectory (same as in panel *A*, but projected onto the *X*-*Y* plane). (*C*, *left panel*) The value *u*(*x*,*y*) calculated with the deformation boundary condition at the membrane-protein interface from the MD profile (*B*) and a random curvature boundary condition, used to produce the starting point for the free-energy-based optimization. (*C*, *right panel*) The final *u*(*x*,*y*) calculated with the CTMD method using the curvature boundary condition that minimizes the membrane-deformation energy penalty (see Mondal et al. (14) for details). Note the agreement between the profile calculated directly from MD (*B*), and that calculated using CTMD (*C*, *right panel*); they are within 0.5 Å RMSD of each other. The added advantage of the CTMD calculation is the evaluation of the energy penalty for the protein-induced membrane-deformation in *u*(*x*,*y*), which in this case is 4.7 kT. The side of a grid square represents the spacing of 2 Å. (This figure is adapted with permission from Mondal et al., 2011 (14). Copyright by Elsevier.) To see this figure in color, go online.

The special properties of multi-TM proteins have major practical consequences with respect to their interaction with the surrounding membrane:

1. An asymmetry is imposed on the HM-dependent membrane deformation, reflecting the radially asymmetric hydrophobic surface. As a result, deformations at specific sites around the protein could be much larger than the average deformation of the membrane, as given in Fig. 2 *A* by the deformations of lipid bilayers of different composition around rhodopsin. These local deformations incur a significant energy cost, because the dependence of the energy cost on the extent of deformations is approximately quadratic (14).
2. Even after such radially asymmetric membrane deformations occur, the HM is not completely alleviated because this would require remodeling in which the membrane adapts to both polar and hydrophobic residues in the same region. A very high energy would be required for the membrane to achieve the local distortion required to accommodate fully the different polarity properties of proximal residues. The remaining exposure to incompatible environments at such loci is termed residual hydrophobic mismatch (RHM), and carries an energy cost (18).

The following sections review the mechanistic impact of these important consequences of hydrophobic mismatch of multi-TM proteins with radially asymmetric hydrophobic surfaces. In particular, we address the membrane remodeling patterns and energetics of the protein-membrane interactions utilizing a quantitative multiscale approach developed recently. The method, named continuum-molecular dynamics (CTMD), combines information from molecular dynamics (MD) simulations of membrane-protein systems with a continuum description of the membrane surrounding them (14) to quantify the membrane remodeling pattern and the RHM.

Importantly, CTMD is shown to make possible the identification of residues at the protein-membrane interface that are most critical to the energy cost of protein-membrane interaction; this is necessary to connect the insights from computation to results from high-resolution experiments. The CTMD calculations have therefore been used as described in the examples below, to reveal an apparently new class of functionally and mechanistically important structural elements that explain the well-known participation of HM in the functional mechanisms of membrane proteins. The published methodological details of the CTMD approach (14) are reviewed briefly below to emphasize the key elements that enable it to accomplish the following:

1. Quantify the radially asymmetric membrane deformations from the results of MD simulations, and
2. Evaluate how the energy cost associated with the corresponding RHM depends both on the conformation of the embedded protein and protein-protein interactions such as oligomerization.

This is followed by specific examples of applications to membrane protein systems that show how the energy cost of the membrane-dependent RHM contributes to essential aspects of the functional mechanisms of multi-TM proteins on the cell surface:

1. The transport efficiency of the prototypical NSS transporter, LeuT, in Example A, and
2. The different patterns of spatial organization of highly similar GPCRs, the *β*1 and *β*2 adrenergic receptors (B1AR and B2AR), in Example B.

With these specific examples, we are able to present in this mini-review some new perspectives on established directions in membrane protein research, including the identification of key residues underlying the mechanistic role of hydrophobic mismatch in complex multi-TM proteins. Although this is presented in the context of the pertinent literature on membrane-protein interactions, NSS transporters, and GPCR oligomerization, we note that a detailed review of these comprehensive topics and the many contributions made to them by a very large number of researchers is well beyond the scope of this focused report.

## Quantifying Radially Asymmetric Membrane Deformations

MD simulations of cell surface proteins embedded in explicit atomistic models of membranes of various compositions have produced comprehensive representations of the structure-function relations and mechanisms of increasing complexity and experimentally verified reliability (see Nobel Lectures in Chemistry, http://www.nobelprize.org/nobel_prizes/chemistry/laureates/2013/). Equally, they yield a detailed representation of the rearrangement of lipid molecules around the embedded protein (16,32,33,36–38), taking into account the conformational properties of the protein that may affect their membrane interactions, such as orientation of individual residues and tilts of TMs (39–44).

From MD trajectories for the various membrane protein systems it is possible to extract the pattern of membrane deformation around the embedded protein (Fig. 2 *B*) by fitting a grid to the phosphate atoms of the lipid molecules over the course of the trajectory, followed by time-averaging and spatial smoothing. This calculation takes into account the lipid-protein interactions locally, thereby incorporating the effect of radial asymmetry of the hydrophobic surface (14). However, MD simulations do not directly provide the energy cost of the membrane deformations, Δ*G*_def_, inherent in the calculated pattern. To be able to quantify Δ*G*_def_ for the deformation resulting from the MD simulation, the CTMD method combines the MD results with a formulation in the framework of the continuum elastic theory of membrane deformations. A complete description of the CTMD methodology is given in Mondal et al. (14), and a standalone software, CTMDapp, which implements the CTMD algorithm, can be downloaded from the Membrane Protein Structural Dynamics Gateway (accessible at http://memprotein.org/resources/servers-and-software).

Briefly described, the theoretical framework of CTMD represents the membrane in terms of a continuum, elastic deformable medium. In such a framework, Δ*G*_def_ is approximated as the sum of energy components from compression-extension, curvature, and surface tension, as

(1) $$\Delta G_{\text{def}}=\frac{1}{2}\int_{\Omega }\left\{K_{a}\frac{\left(2u^{2}\right)}{d_{0}^{2}}+K_{c}{\left(\frac{\partial^{2}u}{\partial x^{2}}+\frac{\partial^{2}u}{\partial y^{2}}-C_{o}\right)}^{2}+\alpha \left[{\left(\frac{\partial u}{\partial x}\right)}^{2}+{\left(\frac{\partial u}{\partial y}\right)}^{2}\right]\right\}d\Omega ,$$

which produce the pattern obtained from the MD simulation. In the above, *K*_*a*_ and *K*_*c*_ are compression-extension and bending moduli, respectively; *α* is the coefficient of surface tension; and *C*_*o*_ represents the spontaneous curvature of the monolayer.

To compute Δ*G*_def_, CTMD uses the results from the MD simulations as boundary conditions in the solution of the Euler-Lagrange differential equations for determining the membrane deformation profile *u*(*x*,*y*) (Fig. 2 *C*):

(2) $$\begin{array}{c} K_{C}\nabla^{4}u-\alpha \nabla^{2}u+\frac{4K_{a}}{d_{0}^{2}}u=0,\\ {u|}_{\Gamma_{\text{in}}}=u_{o}\left(x,y\right),{u|}_{\Gamma_{\text{out}}}=0,{\nabla^{2}u|}_{\Gamma_{\text{in}}}=v_{o}\left(x,y\right),{\nabla^{2}u|}_{\Gamma_{\text{out}}}=0. \end{array}$$

The interactions of the membrane with the protein play a role in this boundary value problem through the boundary conditions at lipid-protein interface, Γ_*in*_. The information obtained from the MD trajectory is the membrane deformation *u*_0_(*x*,*y*) at the lipid-protein interface determined from the time-averaged, spatially smoothed membrane deformation profile calculated from MD (Fig. 2 *B*). This *u*_0_(*x*,*y*) is a boundary condition that captures the effects of both the radial asymmetry of the protein hydrophobic surface and of the conformational adaptation of the protein to the interaction with the membrane. The other boundary condition, *v*_0_(*x*,*y*), is obtained from a self-consistent iteration in the optimization procedure that determines the *v*_0_(*x*,*y*) value that minimizes Δ*G*_def_ formulated as shown in Eq. 1 in terms of *u*(*x*,*y*).

The technical details involved in numerically solving the fourth-order partial-differential equation in Eq. 2, and performing the optimization for a large membrane-protein boundary, are provided in Mondal et al. (14), where the result of optimization is shown to converge to a membrane deformation profile that is very similar to that obtained directly from MD. Note, however, that Δ*G*_def_ cannot be determined by directly plugging *u*(*x*,*y*) from MD (Fig. 2 *B*) into Eq. 1, because the computation of second derivatives from the MD data of *u*(*x*,*y*) can be numerically unstable (14).

CTMD relies on macroscopic parameters (*K*_*a*_, *K*_*c*_, *α*, *C*_*o*_) that describe the physico-chemical properties of the lipid composition. These are available for most commonly known lipids, e.g., for lipids with different tail lengths and with different degrees of unsaturation (45). In addition, *K*_*c*_ can be accurately estimated for mixtures of lipids from relatively inexpensive MD simulations based on the analysis of fluctuations in splay angles for different molecular pairings (46). Importantly, the iterative procedure implemented in CTMD offers an internal consistency check of the macroscopic membrane deformations in the comparison to the pattern obtained from MD, because the resulting deformation patterns are the same only if Eq. 1 is a good representation of the free energy of membrane deformations in the system.

Fig. 2 *A* shows the membrane deformation profiles for rhodopsin embedded in lipids of different thickness—di(C14:1)PC (a lipid with two monounsaturated 14-carbon tails) and di(C20:1)PC (a lipid with two monounsaturated 20-carbon tails). The tendency toward hydrophobic matching is made evident by di(C14:1)PC bilayer thickening on average, and the thicker di(C20:1)PC bilayer becoming thinner on average. The average membrane deformation obtained for each of the membranes surrounding rhodopsin (14) was in good agreement with those inferred from NMR experiments on similar membrane-protein systems (30). However, the large local membrane deformations (e.g., *red regions* in the *left panel* of Fig. 2 *A* and *blue regions* in the *right panel* of Fig. 2 *A*) incur a much larger energy cost than would be expected by simply considering the average membrane deformations. For example, Δ*G*_def_ for di(C14:1)PC was found to be 4.7 kT, much larger than the ∼1.9 kT calculated using the average membrane deformation at the lipid-protein interface (14).

## Calculation of the Residual Hydrophobic Mismatch and its Energy Cost

Local membrane deformations notwithstanding, the hydrophobic matching was revealed to remain incomplete in various multi-TM proteins (14,15,17,18). Such RHM was found for several different membrane-protein systems involving GPCRs and transporters:

1. Rhodopsin in membranes composed of lipids of different tail lengths di(C14:1)PC, di(C16:1)PC, di(C18:1)PC, and di(C20:1)PC (14);
2. Serotonin (5-HT_2A_) receptor in complex with different ligands in a ternary mixture containing SDPC, POPC, and cholesterol (14,16);
3. B1AR and B2AR in POPC/10% cholesterol (15); and
4. Transporter LeuT in POPC bilayer or in a membrane with nativelike 3:1 mixture of POPE and POPG (18).

**Figure 3:**
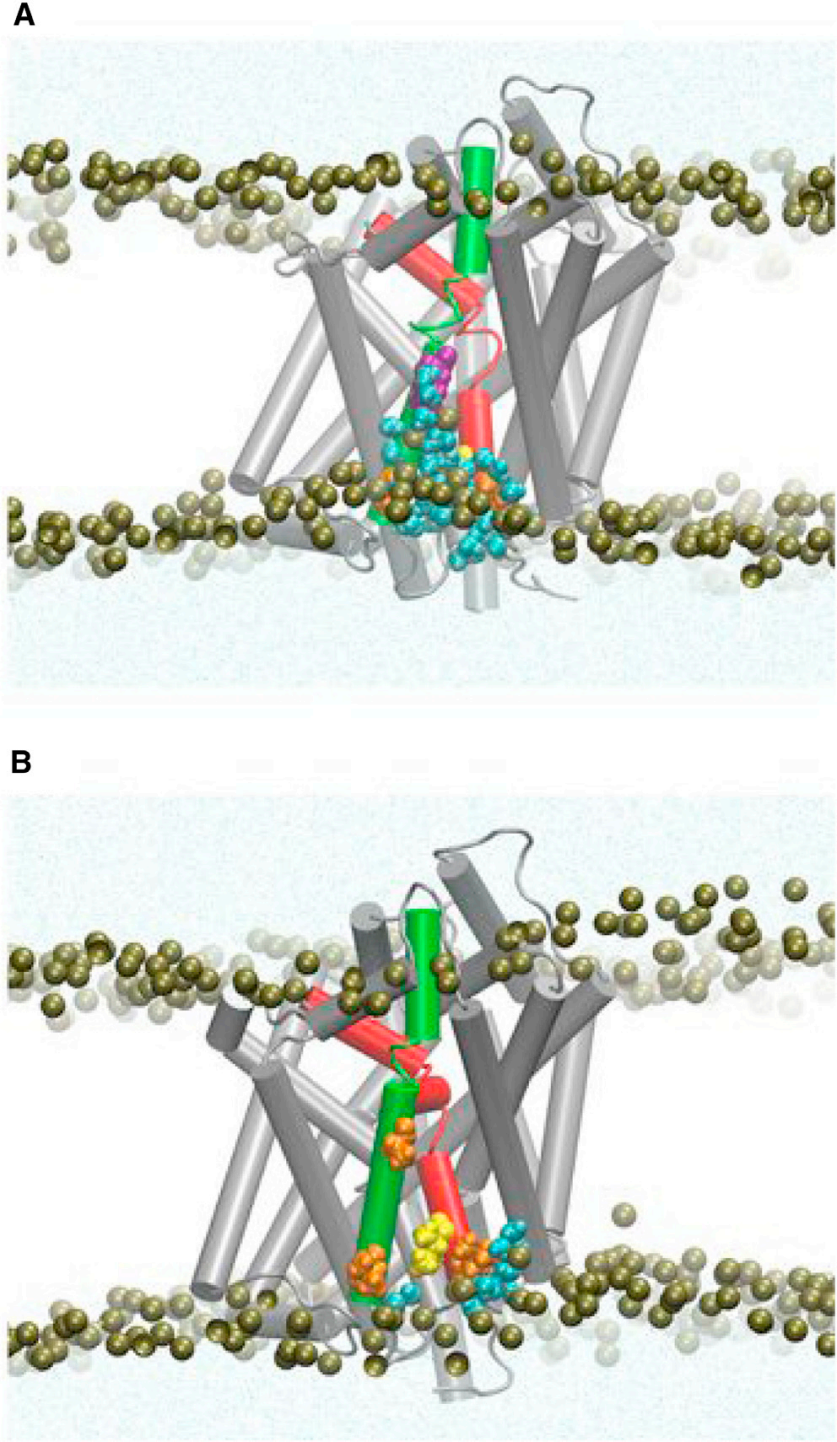
Residual hydrophobic mismatch of a model bacterial transporter, LeuT, in a nativelike POPE/POPG (∼3:1) lipid bilayer. (*A*) A snapshot from the MD trajectory of LeuT in its occluded conformation (see Mondal et al. (18)), illustrating the exposure to water of the hydrophobic residues L12 and L277. (*B*) A snapshot from the MD trajectory of LeuT in its occluded conformation but with K288 mutated to Ala, showing that the mutation removes the water penetration to K288, and reduces the exposure of L12 and L277 to the water. In the two panels, the hydrophobic residues L277 and L12 are highlighted (*orange*), I15 (*yellow*). The residue at position 288 is highlighted (*purple*) for the polar Lys in panel *A*; and (*medium orange*) for the nonpolar Ala in panel *B*. Water molecules within 5 Å of these residues are shown (*cyan* and in *CPK representation*). The membrane is indicated by the phosphates of the two leaflets (*tan*). The corresponding energy cost of RHM is evaluated to be much smaller in the K288A mutant compared to the wild-type LeuT: 1.1 kT at TM1 and 0.2 kT at TM7 of the K288A mutant, compared to 3.2 kT at TM1 and 2.2 kT at TM7 of the wild-type LeuT. (This figure is adapted with permission from Mondal et al., 2013 (18). Copyright by Elsevier.) To see this figure in color, go online.

Fig. 3 *A* shows the incomplete hydrophobic matching, with the example of LeuT embedded in a nativelike mixture of POPE/POPG mixture. The snapshot from the MD trajectory of this system shows how the membrane-facing hydrophobic residues L12 (*orange*) of TM1 and L277 (*purple*) of TM7 are exposed to water.

The incomplete hydrophobic matching at L12 and L277 occurs due to the presence of juxtaposed charged residue K288 (*purple*) in TM7, an adjacency that was shown in Fig. 1 *A*. The local membrane thinning near K288 that results from the local remodeling of the membrane to bring a polar environment closer to the lysine is accompanied by water penetration to this residue. This results in the exposure of the nearby L14 and L277 residues to the water environment, as shown in Fig. 3 *A*. Remarkably, MD simulations of a mutant LeuT with K288 replaced by alanine (K288A) show that water penetration, and the incomplete hydrophobic matching at L12 and L277, were substantially reduced (Fig. 3 *B*).

The incomplete hydrophobic matching described here demonstrates the apparently new concept in the theory of hydrophobic mismatch, which we had termed residual exposure or residual hydrophobic mismatch (RHM) (14,15,18). It should be emphasized that RHM goes beyond the concept of hydrophobic slippage described in Nielsen et al. (24) and Marsh (47). Although RHM can depend on the bulk membrane thickness (14), it is not simply a consequence of a large hydrophobic mismatch between the membrane and the average hydrophobic thickness of the protein. Rather, it arises as described above, from the effect of a particular structural motif(s) in the molecular structures of multi-TM proteins, that is, the adjacent location of polar and hydrophobic residues. The calculations across different membrane-protein systems suggest that the RHM occurs most frequently at membrane-facing regions of the protein where polar and hydrophobic residues are adjacent, such that the membrane is unable to match both polar and hydrophobic residues in the same region (14,15,17,18).

The energy cost of RHM relative to perfect hydrophobic matching, Δ*G*_res_, is proportional to the area of the residues exposed to unfavorable hydrophobic-polar interactions,

(3) $$\Delta G_{\text{res}}=\sum_{r=1}^{R}\sigma SA_{\text{res},r},$$

where *SA*_res,*r*_ is the unfavorably exposed surface area of the *r*^th^ membrane-facing residue; *R* is the total number of membrane-facing residues; and *σ* represents the constant of proportionality. The value for *σ* is based on the transfer energies of residues between polar and hydrophobic media, and taken to be 0.028 kcal/(mol.Å^2^) (48,49) in the calculations presented below. A rapid first evaluation of potential residues creating RHM can be performed as described in Mondal et al. (18), from an x-ray structure (or a model) using the software CTMDapp mentioned above.

*SA*_res,*r*_ can be calculated from the time-averaged solvent-accessible surface areas (SASA), which is a quantity that is routinely extracted from MD trajectories (50,51). For a hydrophobic residue, *SA*_res,*r*_ is the area of the residue that is exposed outside the hydrophobic core of the lipid bilayer, and given by

(4a) $$SA_{\text{res},r}=\mathrm{SASA}\left\{\mathrm{solute}:\mathrm{the}\;\mathrm{protein}+\mathrm{the}\;\mathrm{hydrophobic}\;\mathrm{core}\;\mathrm{of}\;\mathrm{the}\;\mathrm{lipid}\;\mathrm{bilayer}\right\}.$$

For a polar residue, *SA*_res,*r*_ is the area of the residue that is exposed to the hydrophobic core of the lipid bilayer, and given by

(4b) $$SA_{\text{res},r}=\mathrm{SASA}\left\{\mathrm{solute}:\mathrm{protein}\right\}-\mathrm{SASA}\left\{\mathrm{solute:protein}+\mathrm{hydrophobic}\;\mathrm{part}\;\mathrm{of}\;\mathrm{the}\;\mathrm{membrane}\right\}.$$

Certain residues are not included in the calculation of the energy penalty, even if they are membrane-facing. This exemption pertains to interfacial lysine and arginine residues if the mismatch at these residues is alleviated by means of snorkeling (52), and serine and threonine residues if their polar parts form H-bonds with the helix backbone (53). Tryptophan that is preferentially located at the lipid-water interface is also included in this list (54).

This review of the method shows how the calculated effects on, and of, the membrane, as well as their consequences, are evaluated in the quantification of the RHM after the system of membrane/protein has come to an equilibrium in the MD simulation. Therefore, these calculations take into account fully the structural and dynamic properties of the evaluated conformational (or organizational) state of the protein. This quantification of the membrane deformation profile and the RHM is stable for the equilibrated MD trajectory, as indicated by comparison of these properties between different segments of the trajectory. Thus, even for the relatively complicated situation of B2AR oligomers, the comparison shows them to be very similar (15).

## Specific Examples of the Contribution of RHM Energy to Functional Mechanisms of Multi-TM Membrane Proteins

### Example A

#### The energy cost of RHM affects the probability of conformational transitions related to substrate transport by LeuT

Quantification of the RHM for LeuT showed that residue adjacencies in both TM1 and TM7 generate energy penalties much larger than 1 kT (see Table 1 and the legend of Fig. 3 for the values of energy costs), due mostly to the effect of K288 in TM7. Given the purported role of TM1 in the transport mechanism (28), an RHM energy cost for TM1a (i.e., intracellular part of TM1) is striking, because this segment of the transporter was found to undergo a large conformational change during transport (39,40). Specifically, the transformation from outward-open/occluded to inward-open conformation requires a pronounced outward rotation of TM1a, based on evidence from single-molecule FRET (39,40), molecular simulations (28,55), and x-ray crystallography (11,26).

The quantification of RHM (18) from MD trajectories of LeuT that had been shown to correspond well with single-molecule FRET (smFRET) measurements of LeuT dynamics (39,40) shows that this movement of TM1a occurs in the presence of RHM and is affected as described below by the associated energy penalty. In relating the calculations to the dynamic trajectory, the results suggested as well how the RHM may affect the function of the transporter (18). Thus, the total energy cost due to RHM is approximately the same in the outward-open, occluded, and inward-open conformations, but the residues subjected to RHM in TM1a are changing in the dynamic rearrangement (18). Specifically, L12 is involved in RHM only in the outward-open and occluded conformations, but in the inward-open conformation RHM affects L12 as well as I15 (Table 1).

This suggests that the conformational rearrangement of TM1a, that is required for transport, will have to overcome an energy barrier caused by the change in RHM associated with the switch from L12 to L12/I15 of TM1a. Moreover, it explains the experimental observation that K288-to-Ala mutation improves transport rates, because in K288A the RHM at the neighboring L14 and L277 is reduced from 5 kT to only ∼1 kT (Fig. 3). The reduction of the energy barrier is likely responsible for the improved transport rates observed (56) for the mutant construct K288A. This prediction of an energetically more probable transition between the inward-open and occluded conformations of TM1a in the K288A mutant than in WT LeuT can be tested specifically with the same type of smFRET experiments carried out previously (39,40), in which the frequencies of conformational transitions of TM1a are compared in the wild-type and the K288A mutant transporter.

By connecting the local RHM to functional phenotypes, these observations illuminate the role that the concept of RHM can have in facilitating the design of new experiments relying on a specific structural context and mutational strategies. We note, moreover, that the concept of mechanistically significant RHM given here for transport in the prototypical NSS, LeuT, may be helpful in understanding the transport mechanisms of many other transporter proteins, such as the betaine transporter BetP (e.g., see Koshy et al. (13)), some of which share LeuT-like folds (e.g., see Penmatsa and Gouaux (57) and Shi and Weinstein (58)). Indeed, the mechanistic significance of the structural motif of adjacent polar and hydrophobic residues goes beyond transporters, and is next given for another widely studied family of multi-TM proteins: the GPCRs.

### Example B

#### The role of RHM in the modulation of function and spatial organization of GPCRs

Evidence from studies employing different biophysical and biochemical methodologies suggests that GPCRs can form dimers and/ or higher-order oligomers (59–63). However, fundamental aspects of GPCR oligomerization have remained unclear and are currently under intense scrutiny, including the molecular mechanisms driving the spatial organization of GPCRs on the cell surface and the relation of oligomerization to function (16,17,59–61,64,65). We have shown that RHM in GPCR-membrane systems (14,15) explains, for the first time to our knowledge, certain fundamental aspects of the underlying molecular mechanisms. In particular, the energy cost of RHM can rationalize some of the dimerization interfaces observed in x-ray structures of GPCRs (14–17,66,67), explain why the association observed experimentally in cells and membrane systems occurs at specific sets of residues and in a ligand-dependent manner (16), and also how the extent and pattern of oligomerization (15) depends on the receptor subtype, e.g., B1AR versus B2AR (see further below).

The central principles of these explanations are the following:

1. Modes of oligomerization that remove energetically costly membrane interactions should be favorable, and
2. Quantification of the RHM identifies particular protein residues where the energy cost is largest.

The consideration of the RHM in a monomer as a driving force for oligomerization was demonstrated in silico from MD simulations of the spontaneous oligomerization of a prototypical GPCR, the B2AR, in a lipid bilayer composed of the commonly used POPC lipids (15). The simulations were performed at the coarse-grained level, so as to reach ∼20 *μ*s timescales with nine GPCRs in a large membrane patch. The MARTINI force field was employed (68) because it has been shown to perform well for hydrophobic mismatch-related phenomena (68) and was used to study GPCRs such as rhodopsin, including those with similar simulations of spontaneous GPCR oligomerization (69,70).

**Figure 4:**
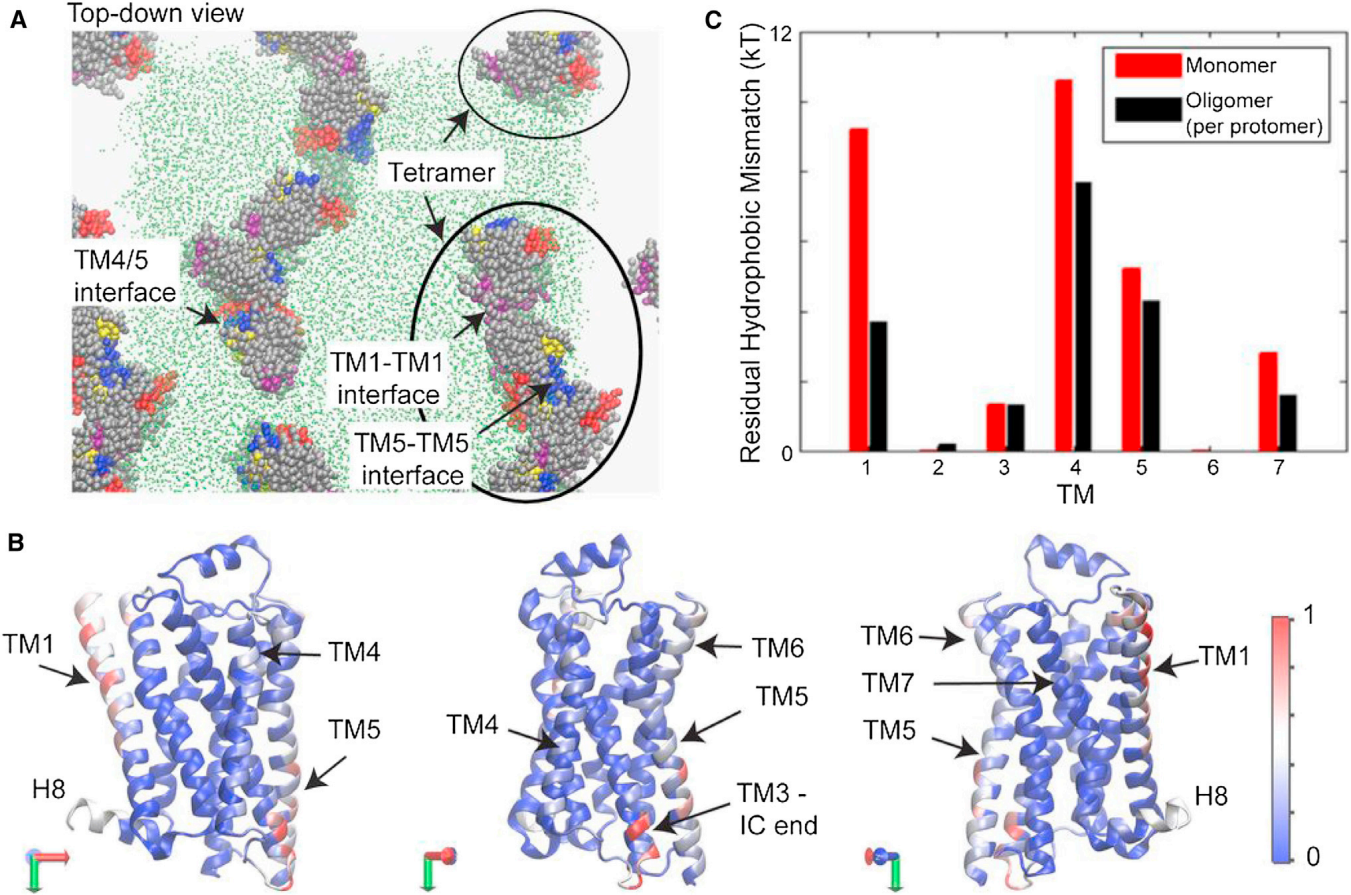
GPCR oligomerization reduces residual hydrophobic mismatch. (*A*) Snapshot from the end of an ∼18-*μ*s MD simulation of B2AR oligomerization in the POPC lipid bilayer, showing the spontaneously evolved higher-order oligomers of B2AR (see Mondal et al. (15)). Note that the panel shows the central simulation cell, and its neighboring periodic replicas. For the sake of clarity, the membrane is shown (*green dots*) in the simulation cell only. Water is also not shown. The proteins are rendered in van der Waals representation. To indicate the orientation of the proteins in these arrays, specific parts of the GPCR proteins are colored as follows: TM1 (*purple*), TM4 (*red*), TM5 (*blue*), TM6 (*yellow*), and the remainder (*silver*). The oligomeric arrays emerging from the simulations are found to involve the typical interfaces such as TM1-TM1, TM5-TM5, and TM4/5. (*B*) The relative frequency with which the different regions of the protein participate in protein-protein interactions during the last 1.4 *μ*sec, shown in a color-coded heat map projected onto a x-ray structure of B2AR (PDB:2RH16) (79). (*Light blue*, *white*, and *red*) Regions involved in protein-protein interactions; (*deep blue*) regions that are not involved in frequent oligomerization contacts during the simulation. The heat map is presented on three different views of the protein to show the entire protein surface. The total number of interactions for each residue is normalized to the maximum frequency of interactions for all residues during this time period. (*C*) The average energy cost of residual hydrophobic mismatch (RHM) for each TM of B2AR embedded in the POPC membrane bilayer, calculated for a protomer in the oligomeric arrays (in *black*), and compared to that calculated for the monomeric protein (in *red*). This figure is adapted from Mondal et al. (15). To see this figure in color, go online.

After several events of association-dissociation observed in the long trajectory, spontaneously formed arrays of GPCR oligomers emerged in the simulated system, and remained stable over the time course of the simulation (Fig. 4 *A*). All these oligomeric arrays involved specific sets of residues, shown in the heat map of Fig. 4 *B*. Fig. 4 *C* compares the RHM in the oligomers to that calculated in the monomers from the early part of the simulation, and reveals that the RHM decreases upon oligomerization. Moreover, the identification of RHM in specific regions of the unassociated protomers marks the putative TM-TM interfaces, and the atomistic detail suggests specific modes of validation of the oligomerization drive using mutational strategies.

**Figure 5:**
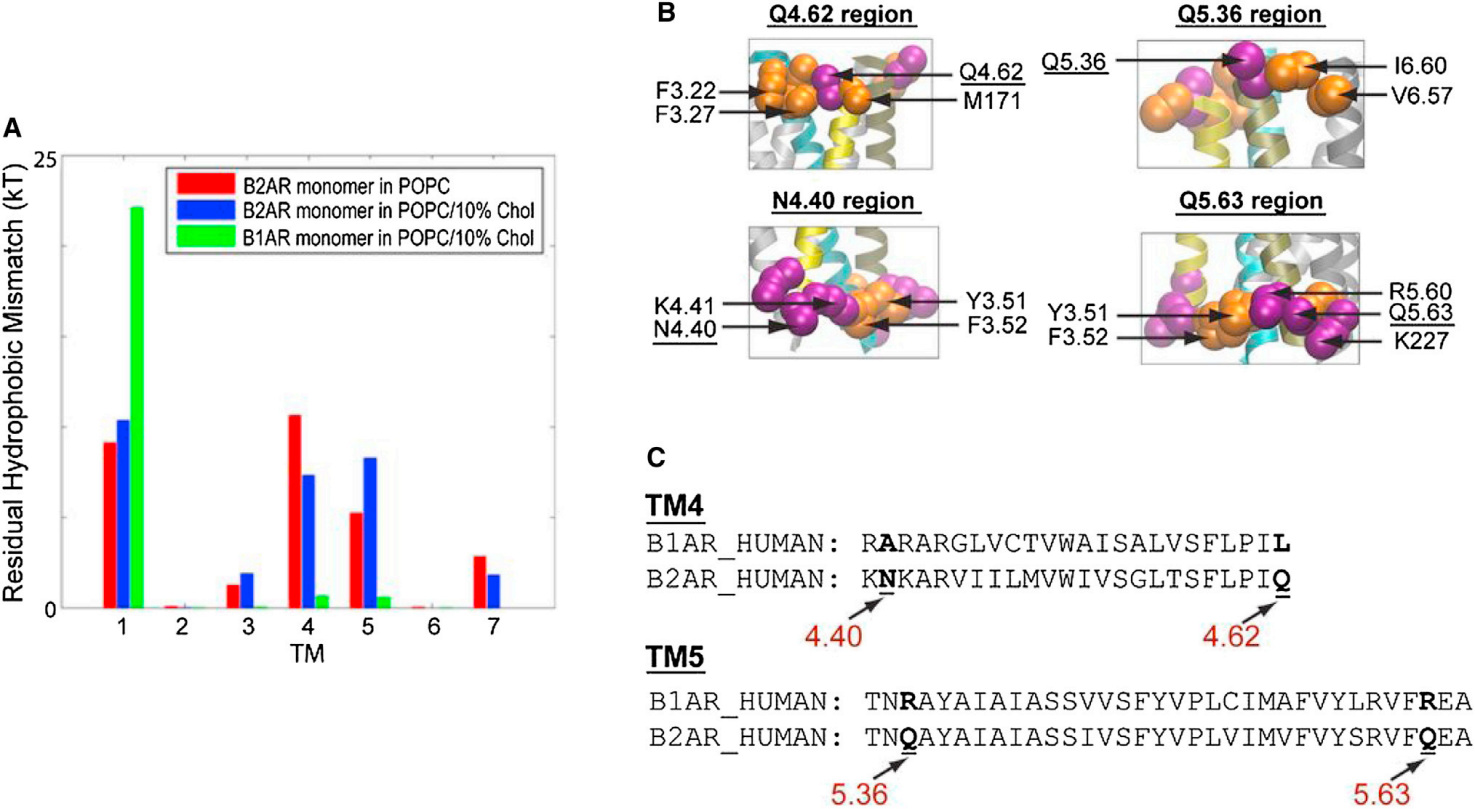
Comparison of residual hydrophobic mismatch of B2AR and B1AR. (*A*) Average energy cost of residual hydrophobic mismatch for each TM of a B2AR monomer embedded in POPC/10% Cholesterol bilayer (*blue bars*), compared to the results for the B2AR monomer in POPC, without cholesterol from Fig. 4*C* (*red bars*), and to results for the highly homologous B1AR monomer in the POPC/10% cholesterol bilayer (*green bars*). (*B*) The structural context of the incomplete hydrophobic matching observed at the TM4/TM5 interface of B2AR; such incomplete matching is not observed for B1AR. Adjacent polar (*purple*) and hydrophobic (*orange*) residues occur in TM4/TM5 of B2AR. (*C*) Structure-based sequence alignment of B1AR versus B2AR (80) for TM4 and TM5, with the loci where the two homologous GPCRs differ in terms of hydrophobic character (indicated in *boldface*). This figure is adapted from Mondal et al. (15). To see this figure in color, go online.

Comparison of Fig. 4, parts *B* and *C*, reveal significant RHM at diametrically opposite regions of the B2AR molecule. Interpreting the associated energy penalties as oligomerization drivers, this observation suggests that, as a consequence of the lipid-protein interactions, the B2AR will organize spatially not only in dimers but also in higher-order oligomers with interfaces at those diametrically opposite regions. This pattern of RHM was indeed observed in POPC membrane containing 10% cholesterol (Fig. 5 *A*), consistent with the prediction of higher-order oligomers from FRET monitoring of B2AR oligomerization (71,72). Interestingly, a different oligomerization pattern was predicted for the B1AR compared to the B2AR (72), and the quantification of RHM provides a mechanistic explanation based on different modes of interaction with the membrane. Thus, despite the high sequence homology between the two adrenergic receptors, results in Fig. 5 *B* show that the RHM for B1AR is predominantly localized at TM1, but large RHM values are found at both TM1 and TM4/TM5 for B2AR.

Considering the RHM in a monomer as a driving force for oligomerization, these findings predict an oligomerization pattern that is consonant with the FRET data, suggesting that B1AR predominantly forms dimers, whereas B2AR forms tetramers as well (72). Fig. 5 *B* highlights the local structure of the region in TM4/TM5 where a significant RHM was calculated for B2AR, but not for B1AR. It is evident that for B2AR, the RHM in this region is associated with an adjacency of hydrophobic and polar residues, but the structure-based sequence comparison in Fig. 5 *C* shows no such indication for B1AR. The hydrophobic character of corresponding residues in this region differs in the two otherwise highly homologous GPCRs.

It is important to emphasize that as the overall conformation of a GPCR can change upon interaction with ligands that differ pharmacologically or structurally, the protein-membrane interaction will change, and so will the pattern of RHM (14,16). Activation of class A GPCRs, for example, is associated with an outward movement of TM6, which changes the lipid-protein interface (16). Indeed, the calculations for interactions of the serotonin 5HT_2A_ receptor with the agonist 5HT versus the partial agonist LSD versus the inverse agonist Ketanserin (14,16) have shown distinct differences in the pattern of RHM. This consideration explains how the oligomerization interface for GPCRs (e.g., the commonly observed TM4/TM5 interface) may be sensitive to ligands (in fact, to our knowledge, this is the first molecular-level explanation for this pharmacologically important experimental observation) (59).

Because crystallization conditions are different from those used in the study of GPCR function experimentally or with simulations in bilayers, it is noteworthy that the concept of RHM-driven spatial organization could be used as well to explain the formation of lateral contacts in crystallographically obtained arrays of GPCRs (66,67). Thus, in a recent computational study investigating the molecular mechanisms of GPCR crystallization in the lipidic cubic phase, the dimerization interfaces suggested from the pattern of RHM in the A_2A_ adenosine receptor were found to be in excellent agreement with the dimerization interfaces observed crystallographically (66).

## Conclusion

The function of complex proteins at the cell membrane surface involves interactions with the membrane and with a variety of other proteins in their environment. For the most part, however, the molecular mechanisms underlying the functions of such membrane proteins have been investigated separately from the properties of surrounding membranes (73). With the lipid bilayer often viewed as not more than a relevant medium in which the protein is reconstituted in experimental and computational setups, fundamental steps of the function of various multi-TM proteins are being studied with the aim of identifying residue-level motifs of mechanistic importance. However, the considerations highlighted in this mini-review suggest that the mechanistic elements of function will depend on the modulation of protein-membrane interactions by ligand-binding properties, conformational changes, oligomerization patterns, and other modulators of hydrophobic mismatch. Indeed, the membrane proteins have different conformational and organizational states reflecting their structures and interactions, but their properties and functional mechanisms have much to do with the equilibrium distribution between these states and/or the transition between these states.

In this equilibrium distribution, the energy spectrum of membrane-protein interactions (such as hydrophobic mismatch) is a key determinant. Therefore, we emphasize that although the conformational states of proteins are dependent on a number of key intrinsic factors—from sequence to folding—a rapidly growing literature on this subject makes it clear that for proteins embedded in membranes, the equilibrium between the different conformational and organizational states, and/or transitions between them, involve energy components that depend on residue-level interactions with the membrane. Mechanistic investigations must, therefore, address the properties of the lipid membrane and the way these affect the energetics of the molecular mechanisms. In turn, the considerations presented here indicate that the investigation of biophysical properties of lipid membranes must be enriched by the context of interactions with the types of proteins they affect, which are usually more complex than the simple single-helical constructs commonly employed in such studies. The examples presented in this work for multi-TM proteins that represent large membrane protein families show how the needed attention can be accorded to capturing quantitatively the effects produced by the interaction between phospholipid membranes with structurally and dynamically complex proteins.

The methodological advances and computational tools discussed in this mini-review should facilitate the concerted representation of properties and mechanisms of multi-TM proteins in their membrane environment, both quantitatively and at the detailed residue-level necessary to make contact with the resolution of experimentation that protein studies have been achieving. In particular, the review identifies a mechanistically important structural occurrence in the multi-TM proteins, that is, adjacent membrane-facing polar and hydrophobic residues, as a major factor in the well-known functional role of hydrophobic mismatch. This advances the understanding of hydrophobic mismatch (21,24,47,74–77) to the level of molecular structures of complex multi-TM proteins. Such a detailed understanding of lipid-protein interactions should aid the efforts to represent in computational models the molecular mechanisms of membrane proteins, while taking advantage of the increasingly available structural and functional data at the residue-level resolution. For both GPCRs and transporters, for example, the recent rapid pace of data acquisition from x-ray diffraction studies (12,13,26,41,42,57,78–81) has brought to light a large amount of structural information that can serve to extract important mechanistic insights. As provided here, such analysis must integrate the role of structural elements involved in the modulatory role of lipid-protein interactions, and give careful and quantitative consideration to the effects of ligands and protein-protein interactions in the dynamic rearrangements driven by the membrane environment.

## Figures and Tables

**Table 1:** Energy penalty of RHM (in kT) at L12 and I15 of TM1 and L277 of TM7 for the outward-open, occluded, and inward-open conformations of the transporter

	Outward-open	Occluded	Inward-open
L12 (TM1)	3.3	3.1	1.4
I15 (TM1)	<1	<1	2.0
L277 (TM7)	3.0	3.4	2.9

These calculations were performed (see Mondal et al. (18) in corresponding MD trajectories of LeuT in POPC, which had been developed in conjunction with smFRET experiments (39,40)).
